# A Comprehensive Assessment of Quality of Antimalarial Medicines in Mainland Tanzania: Insights from Five Years of Postmarket Surveillance

**DOI:** 10.4269/ajtmh.24-0145

**Published:** 2024-10-01

**Authors:** Eulambius M. Mlugu, Jacob Mhagama, Damas Matiko, Siya Agustine, Moses Nandonde, Emmanuel Masunga, Peter P. Kunambi, Raphael Zozimus Sangeda, Yonah H. Mwalwisi, Adam Fimbo

**Affiliations:** ^1^ Department of Pharmaceutics and Pharmacy Practice, Muhimbili University of Health and Allied Sciences, Dar es Salaam, Tanzania;; ^2^ Tanzania Medicines and Medical Devices Authority, Dodoma, Tanzania;; ^3^ Department of Clinical Pharmacology, School of Biomedical Sciences, Campus College of Medicine, Muhimbili University of Health and Allied Sciences, Dar Es Salaam, Tanzania;; ^4^ Department of Pharmaceutical Microbiology, Muhimbili University of Health and Allied Sciences, Dar es Salaam, Tanzania

## Abstract

Sustainable access to high-quality antimalarial medicines is pivotal to achieving universal and effective malaria control. Poor-quality antimalarial medicines are prevalent in sub-Saharan Africa, impeding malaria control initiatives and claiming the lives of many children. Regular monitoring of the quality of antimalarial medicines is crucial to ensure the quality of service to the community. A cross-sectional study using a postmarket surveillance (PMS) approach was conducted from 2019 to 2023. Samples were collected from the port of entry, local manufacturers, and various distribution outlets in 15 regions of mainland Tanzania. The samples were subjected to tier 1 evaluation, comprising a product information review (PIR) and identification using the Global Pharma Health Fund-Minilab^®^ techniques. Samples that failed the identification tests and 10% of the samples from distribution outlets that passed the tests were subjected to confirmatory testing (tier 2), which included assays, related substances, dissolution, and sterility per the pharmacopeial monographs. During five annual PMSs, 2,032 antimalarial samples were collected and subjected to quality tests. All samples complied with the standard specifications for identity, dissolution, related substances, sterility, physical evaluation, disintegration, and assay. A total of 292 (55.5%) tested samples failed the PIR evaluation, with incomplete package information in leaflets contributing to 64.7% of all deviations. Antimalarial medicines circulating in the mainland Tanzanian market meet expected quality standards. Continuous monitoring of the quality of antimalarial medicines is recommended.

## INTRODUCTION

Malaria continues to pose significant global health challenges, with an estimated 249 million cases and more than 600,000 associated deaths reported in 2022.^1^ Sub-Saharan African countries accounted for 95% of all malaria cases and deaths worldwide that year.^1^ In particular, Tanzania remains a focal point for malaria, with an overall prevalence of 8% in 2022.^2^ Alarmingly, Tanzania ranks fourth among the four countries, contributing to more than half of global malaria-associated deaths in 2022.^1^ In alignment with the global Sustainable Development Goal (SDG) number 3.3, which aims to eradicate malaria epidemics by 2030,^3^ Tanzania has set ambitious targets through its 2021–2025 malaria strategic plan. The plan seeks to mitigate malaria-related morbidity and mortality by promoting universal access to early diagnosis, prompt treatment, and preventive therapies for vulnerable groups.^4^ Achieving these objectives requires sustainable access to high-quality, safe, and efficacious antimalarial medicines.

Antimalarial medicines have revolutionized malaria control by offering significant efficacy and strengthening disease management. However, a major impediment arises from substandard or falsified antimalarial medicines. These poor-quality medications have not only undermined malaria control initiatives but have also cost the lives of numerous children. A modeling study estimated that more than 122,000 children under the age of 5 years in sub-Saharan Africa die annually because of falsified or substandard antimalarial medicines alone.^5^

Substandard antimalarials contribute to increased costs for patients and the healthcare system. Between 2005 and 2015, substandard and falsified medicines, including antimalarials, incurred a total cost of US$13.65 million in Tanzania.^6^ Exposure to poor-quality antimalarial medicines can lead to inadequate treatment and can foster the development of drug resistance.^7^ The emergence of artemisinin resistance in the Great Mekong region has been attributed to falsified and substandard antimalarial medicines.^8^^,^^9^ This emphasizes the importance of ensuring the quality of antimalarial medicines to prevent the development of resistance.

The prevalence of substandard and falsified antimalarial medicines is widespread in malaria-endemic countries. A systematic review revealed that poor-quality antimalarial medicines are especially frequent in African and Asian countries,^10^ with an overall prevalence of 19.1%.^11^ Instances of poor-quality artemisinin-based combination therapy were reported in Ghana, Togo,^12^ and Malawi.^13^ A survey conducted in Afghanistan identified substandard concentrations of quinine and sulfadoxine/pyrimethamine (SP) at a rate of 32%.^14^ A recent study from Bukavu, Democratic Republic of Congo, reported a high burden of poor-quality antimalarial medicine, with 8.3% of quinine samples containing an active ingredient other than quinine.^15^

Previous studies in Tanzania have highlighted high rates of poor-quality antimalarial medicines on the market.^16^^–^^20^ However, these studies focused solely on products from private distribution outlets and overlooked other crucial points in the supply chain. This study addressed this gap by encompassing the entire medicine distribution chain, including importers at the port of entry, domestic manufacturers, and public and private distribution outlets from 15 regions of mainland Tanzania. A similar study more than 5 years ago identified poor-quality antimalarial medicines in the market,^21^ emphasizing the imperative for continuous surveillance.

Routine surveillance is crucial for building community confidence and ensuring that medicines circulating in the market meet expected quality standards, ultimately delivering the anticipated benefits. This study also provides evidence for stakeholders involved in malaria control as it will enable them to make informed decisions. Therefore, we present the findings of five annual postmarket surveillance (PMS) assessments regarding the quality of antimalarial medicines circulating in the Tanzanian market.

## MATERIALS AND METHODS

### Study design and setting.

A descriptive cross-sectional design was used to examine the quality of antimalarial medicines available in the market. For 3 years of PMS (2019, 2021, and 2022), samples were collected from the port of entry and domestic manufacturers. For 2 years (2020 and 2023), samples were collected from ports of entry, domestic manufacturers, and distribution outlets. The survey was conducted in 15 regions of mainland Tanzania. These regions are Arusha, Dar es Salaam, Dodoma, Coast, Iringa, Kagera, Katavi, Kilimanjaro, Mara, Mbeya, Morogoro, Mwanza, Mtwara, Njombe, and Tanga. The regions bordering neighboring countries, those with hot and humid climates or cold climates, and those with mining and fishing activities were chosen based on predefined criteria. The entire medicine distribution chain, including the National Medical Stores Department (MSD), domestic manufacturers, referral/regional hospitals, district hospitals, faith-based hospitals, private hospitals, wholesale pharmacies, retail pharmacies, and accredited drug distribution outlets (ADDOs) were included in each region.

### Sampling.

The study targeted all products available on the market, including artesunate, quinine, quinine syrup, artemether/lumefantrine (ALu) tablets, artemisinin-mefloquine tablets, sulfadoxine-pyrimethamine, sulfamethoxypyrazine-pyrimethamine tablets, and artemisinin-piperaquine tablets. The collection of samples at various levels of the distribution channels was based on the sampling plans developed. The sampling plans included detailed information on the sampling sites at the regional and district levels, product names, number of brands to be collected, dosage forms, strength, and pack size. Samples were collected from governments and private and faith-based facilities and were divided into two levels. Level 1 was regarded as the highest distribution chain level, including importers/wholesalers, manufacturers, and the MSD. The second level consisted of various dispensing outlets, including retail pharmacies, ADDOs, hospitals, health centers, and dispensaries. In each region, samples were collected from a regional hospital (public), one private hospital, private pharmacies, and the MSD where applicable. At the district level, district hospitals, health centers, dispensaries, and ADDOs were the collection sites. One batch and one brand were collected for each medicine at each collection site.

### Sample collection.

Samples were collected according to standard operating procedures by trained medicine inspectors from the Tanzania Medicines and Medical Device Authority (TMDA) and local government authorities. The samples were collected in their original containers or packages, and details of the collected samples were recorded on the sample collection form. Each sample was coded to ensure traceability. Coded samples and their respective collection forms were stored and sealed in labeled sampling envelopes/plastic bags. A sufficient number of samples and units were collected to enable quality laboratory analysis of the prescribed test parameters. The units collected depended on the type of formulation (i.e., tablets and capsules, 100 tablets per brand per batch; injections, 40 vials per brand per batch; and suspension and syrups,10 bottles per brand per batch). The study survey excluded antimalarial medicines used in clinical trial phases I, II, and III, expired medicines, and those whose remaining shelf life was less than 6 months. Unregistered medicines were promptly removed from the market when discovered during the PMS; they subsequently underwent quality testing, and legal actions were taken as necessary. Samples from public facilities, including MSDs and government hospitals, were made available free of charge. The collected samples were stored according to the manufacturer’s recommended conditions at TMDA zone offices before being transported to the TMDA sub-headquarters for quality evaluation.

### Physical evaluation.

Before laboratory analysis, samples collected from the distribution outlets were subjected to a product information review (PIR) to assess the information in the primary and secondary packages. The parameters checked during the PIR included, but were not limited to, the product brand and generic names, dosage form and strength, name and address of the manufacturer, batch number, physical appearance, manufacturing and expiration dates, language, indication, and storage instructions in comparison to what had been submitted during market authorization. All the samples were subjected to physical evaluation. The tablets were checked for shape, size, brittleness, physical damage (capping and chipping), mottling, odor, altered surface (coating and swelling), color, embossing, debossing, color, particulate matter, and clumping. The injectable solutions were checked for clarity, particulates, turbidity, and color.

### Sample screening and testing.

Sample screening was performed at the TMDA’s WHO-Prequalified Medicine Testing Laboratory. Disintegration tests were performed on all solid dosage forms according to the Global Pharma Health Fund-Minilab^®^ (GPHF) manual. Product identification and determination of the possible presence of degradants in the dosage form were performed semi-quantitatively using thin-layer chromatography (TLC) according to GPHF procedures (tier 1). The standards used were primary reference standards obtained from the European Directorate for Quality of Medicine, International Chemical Reference Substance, and US Pharmacopeia suppliers. All samples that failed or had questionable identification tests and 10% of the samples from distribution outlets that passed the identification tests were subjected to confirmatory testing (tier 2). Confirmatory tests included assays, related substances, dissolution, and sterility. Confirmatory tests were performed for artesunate injection, artemether injection, and Alu tablets according to the International Pharmacopeia (IP).^22^ Quinine tablets and syrup in-house methods were modified according to British pharmacopeial monographs.

### Outcome variables.

Study outcomes included quality parameters of the drugs, including TLC identification, PIR, assay, related substances, and dissolution tests. In the PIR, the availability and information provided on the package information leaflet were evaluated against the TMDA labeling guidelines,^23^ and the approved product information was captured in the TMDA Regulatory Information Management System. Visual inspection was performed by comparing the appearance of the dosage form with that of the registered product. The coated tablets were allowed to disintegrate in distilled water at 37°C for less than 30 minutes. For the TLC identification tests, spots between the test and reference solutions were compared in terms of color, shape, size, intensity, and retardation factor (Rf). A test sample was considered to have failed if its Rf value was greater or less than 10% of that of the standard sample. In addition, a sample was considered to have failed if the color intensity of the spot was less than that of a reference spot containing 80% of the stated amount of active principle (API). The assay results were evaluated according to the recommended standards of the IP, whereby concentrations between 95% and 105% of the active ingredients for quinine and between 90% and 110% for artemether, lumefantrine, and artesunate were considered acceptable. For fixed-combination therapies, a sample was considered to have failed if one or more of its component APIs did not meet the pharmacopeial specifications.

## STATISTICAL ANALYSES

Data analysis was performed using STATA 15 (College Station, TX), and categorical variables are summarized as numbers, frequencies, and proportions. GraphPad Prism v. 8.4 for Windows (GraphPad, La Jolla, CA) was used for graphical presentation.

## RESULTS

Antimalarial samples (*n* = 2,032) were collected and tested for quality from June 2019 to December 2023. The majority of the samples (1,506 [74.2%]) were collected from the port of entry, and only 526 (35.8%) were collected from distribution outlets. Artemether/lumefantrine contributed to 74% of collected samples from all sources. The numbers of samples collected from the two sources for each year are listed in Table 1.

**Table 1:** Number of samples collected from the POE and distribution outlets

Year		2019	2020		2021	2022	2023		Total
SN	Medicine	POE and Domestic Manufacturers	POE and Domestic Manufacturers	Distribution Outlets	POE and Domestic Manufacturers	POE and Domestic Manufacturers	POE and Domestic Manufacturers	Distribution Outlets	
		*n* (%)	*n* (%)	*n* (%)	*n* (%)	*n* (%)	*n* (%)	*n* (%)	*N* (%)
1.	Artemether/Lumefantrine Tablets	247 (85.5)	200 (70.7)	84 (53.2)	401 (75.6)	292 (83)	45 (86.5)	234 (63.6)	1503 (74)
2.	Artesunate Injection	22 (7.6)	45 (15.9)	29 (18.4)	69 (13)	34 (9.6)	0 (0)	88 (23.9)	287 (14.1)
3.	Artemether Injection	4 (1.4)	8 (2.8)	0 (0)	0 (0)	0 (0)	1 (1.9)	46 (12.5)	59
4.	Artesunate-Mefloquine	0 (0)	1 (0.4)	0 (0)	0 (0)	0 (0)	0 (0)	0 (0)	1 (0.05)
5.	Artemisinin-Piperaquine	0 (0)	0 (0)	–	2 (0.4)	0 (0)	0 (0)	0 (0)	2 (0.1)
6.	Quinine Tablets	2 (0.7)	7 (2.5)	30 (19)	0 (0)	5 (1.4)	0 (0)	0 (0)	44 (2.2)
7.	Quinine Syrup	0 (0)	0 (0)	15 (9.5)	0 (0)	0 (0)	0 (0)	0 (0)	15 (0.7)
8.	Dihydroartemisinin-Piperaquine	0 (0)	14 (4.9)	0 (0)	8 (1.5)	0 (0)	6 (11.5)	0 (0)	28 (1.4)
9.	Sulfadoxine-Pyrimethamine	5 (1.7)	3 (1.1)	0 (0)	37 (7)	13 (3.7)	0 (0)	0 (0)	58 (2.9)
10.	Sulfamethoxypyrazine-Pyrimethamine	9 (3.1)	5 (1.8)	0 (0)	13 (2.5)	8 (2.3)	0 (0)	0 (0)	35 (1.7)
Total		289	283	158	530	352	52	368	2,032

POE = port of entry; SN = serial number.

**Figure 1.**
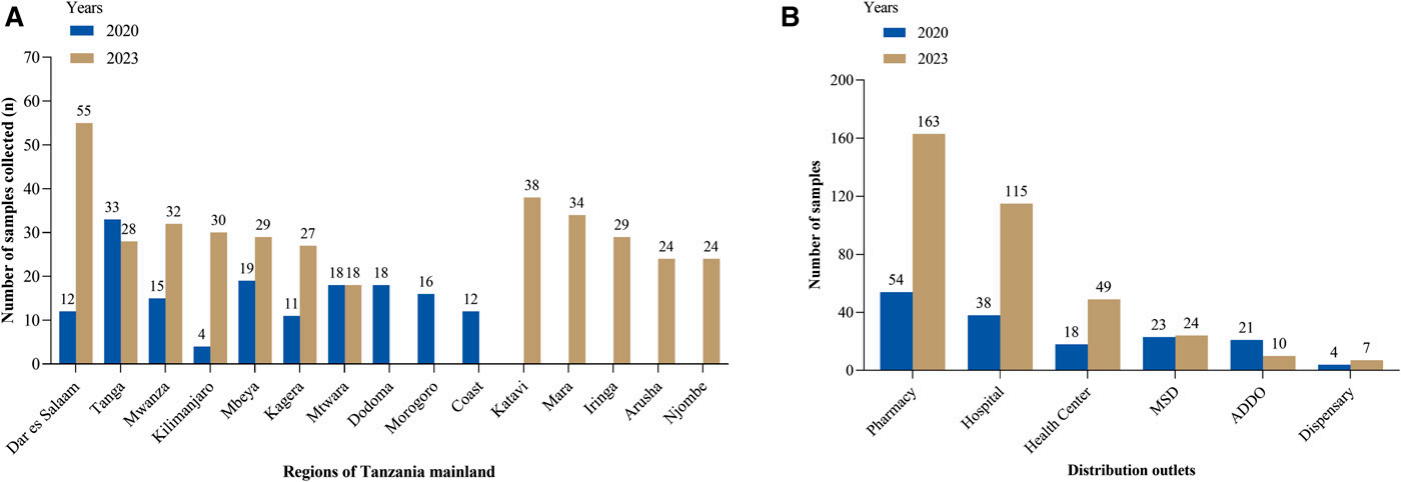
(**A**) Number of samples collected from each region and (**B**) from different distribution outlets. ADDO = accredited drug distribution outlet; MSD = National Medical Stores Department.

Of the total collected samples, 8.5% (172/2,032) were domestically manufactured, and most were imported from India. All samples from the port of entry were collected in the Dar es Salaam region where the main port is located. Samples from distribution outlets were collected from 15 regions. Most samples from the distribution outlets were collected from the Tanga and Dar es Salaam regions, whereas the fewest samples were collected from the Dodoma, coastal, and Morogoro regions (Figure 1A). Community pharmacies contributed to most of the collected samples, whereas the lowest amount was collected from dispensaries (Figure 1B) in both years. The number of each medicine collected from each region is presented in Supplemental Table 1.

### Product information review.

**Figure 2.**
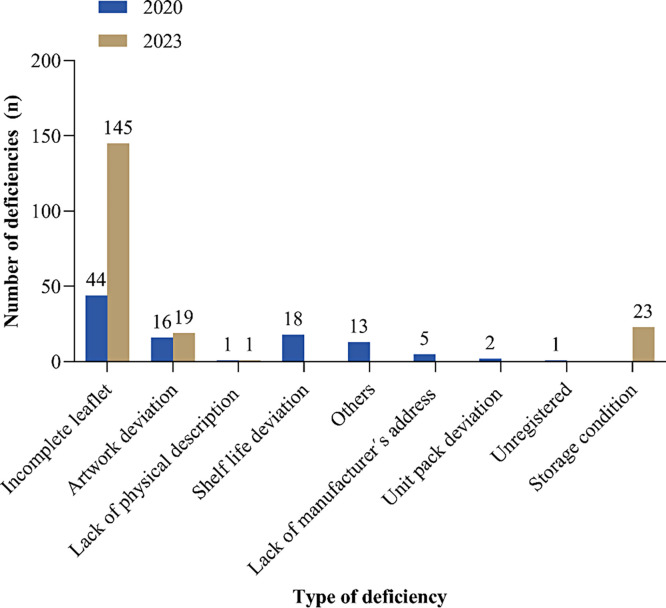
Deficiencies identified among surveyed antimalarial medicines for 2 different years.

All samples collected from medicine distribution outlets were subjected to PIR. Of the 526 samples, 292 (55.5%) failed PIR evaluation. Among the samples that failed PIRs, 61% (178/292) were ALu tablets and 27 (16.1%) were artesunate injection samples. Incomplete package information leaflets were identified in 36% (189/526) of the samples, followed by product artwork deviation in 6.7% (35/526). Figure 2 shows the types and number of samples with deviations for 2020 and 2023.

### Visual evaluation and disintegration test.

All samples subjected to visual assessment met the standard requirements. They all had uniform color, size, and shape, with no observed particulate matter or physical damage corresponding to the registered products. All oral solid dosage forms subjected to disintegration tests complied with requirements in the GPHF Minilab manual (less than 30 minutes). The median disintegration time of the ALu tablets was 4 (1–8) minutes, whereas that of the quinine tablets was 5 (3–7) minutes.

### Minilab identification test.

All samples subjected to TLC screening had comparable spots between the test and reference solutions in terms of color, shape, size, intensity, and Rf values, which complied with standard regulatory requirements. Table 2 shows the screening results for samples collected from port of entry and domestic manufacturers, whereas Table 3 shows the results for samples collected from distribution outlets.

**Table 2:** Screening of samples from POE and domestic manufacturers

Year		2019			2020			2021			2022			2023		
SN	Medicine	Total Screened	Pass	Fail	Total Screened	Pass	Fail	Total Screened	Pass	Fail	Total Screened	Pass	Fail	Total Screened	Pass	Fail
		*n*	*n* (%)	*n* (%)	*n*	*n* (%)	*n* (%)	*n*	*n* (%)	*n* (%)	*n*	*n* (%)	*n* (%)	*n*	*n* (%)	*n* (%)
1.	Artemether/Lumefantrine Tablets	247	247 (100)	0 (0)	200	200 (100)	0 (0)	401	401 (100)	0 (0)	292	292 (100)	0 (0)	45	45 (100)	0 (0)
2.	Artesunate Injection	22	22 (100)	0 (0)	45	45 (100)	0 (0)	69	69 (100)	0 (0)	34	34 (100)	0 (0)	0	–	–
3.	Artemether Injection	4	4 (100)	0 (0)	8	8 (100)	0 (0)	0	–	–	0	–	–	1	–	–
4.	Artesunate-Mefloquine	0	–	–	1	1 (100)	0 (0)	0	–	–	0	–	–	0	–	–
5.	Artemisinin-Piperaquine	0	–	–	0	–	–	2	2 (100)	0 (0)	0	–	–	0	–	–
6.	Quinine Tablets	2	2 (100)	0 (0)	7	7 (100)	0 (0)	0	–	–	5	5 (100)	0 (0)	0	–	–
7.	Quinine Syrup	0	–	–	0	–	–	0	–	–	0	–	–	0	–	–
8.	Dihydroartemisinin-Piperaquine	0	–	–	14	14 (100)	0 (0)	8	8 (100)	0 (0)	0	–	–	6	6 (100)	0 (0)
9.	Sulfadoxine-Pyrimethamine	5	5 (100)	0 (0)	3	3 (100)	0 (0)	37	37 (100)	0 (0)	13	13 (100)	0 (0)	0 (0)	–	–
10.	Sulfamethoxypyrazine-Pyrimethamine	9	9 (100)	0 (0)	5	5 (100)	0 (0)	13	13 (100)	0 (0)	8	8 (100)	0 (0)	0 (0)	–	–
Total		289	289	0	283	283	0	530	530	0	352	352	0	52	52	0

POE = port of entry; SN = serial number.

**Table 3:** Screening of samples from distribution outlets

Year		2020			2023		
SN	Medicine	Total Screened	Pass	Failed	Total Screened	Pass	Failed
		*n*	*n* (%)	*n* (%)	*n*	*n* (%)	*n* (%)
1.	Artemether/Lumefantrine Tablets	84	84 (100)	0 (0)	234	234 (100)	0 (0)
2.	Artesunate Injection	29	29 (100)	0 (0)	88	88 (100)	0 (0)
3.	Artemether Injection	0	–	–	46	46 (100)	0 (0)
4.	Artesunate-Mefloquine	0	–	–	0	–	–
5.	Artemisinin-Piperaquine	0	–	–	0	–	–
6.	Quinine Tablets	30	30 (100)	0 (0)	5	5 (100)	0 (0)
7.	Quinine Syrup	15	15 (100)	0 (0)	0	–	–
8.	Dihydroartemisinin-Piperaquine	0	–	–	0	–	–
9.	Sulfadoxine-Pyrimethamine	0	–	–	0	–	0 (0)
10.	Sulfamethoxypyrazine-Pyrimethamine	0	–	–	0	–	0 (0)
Total		158	158	0	368	368	0

SN = serial number.

### Laboratory confirmatory testing.

A total of 51 samples, which is 10% of the samples collected from the medicine distribution outlets (*n* = 526), were subjected to confirmatory tests including assays, dissolution, related substances, and sterility for injections. These included mono-components of artesunate injection (*n* = 15), artemether injection (*n* = 6), quinine suspension (*n* = 2), quinine tablets (*n* = 3), and a fixed-dose combination of ALu (*n* = 25). For the fixed combination, the tests were done for each component.

After the dissolution tests, the median artemether content was 74% (range: 71–80), and all samples were above 70% after 45 minutes. In addition, the median lumefantrine content was 66% (range: 62–73%), and all samples were above 60% after 45 minutes, complying with IP requirements. The median quinine content was 75%, and none of the samples had below 70% after 45 minutes per standard requirements. No impurity peaks were detected for all the tested samples, complying with the pharmacopeial requirements for related substances. Artesunate and artemether injections complied with standard requirements of sterility.

**Figure 3.**
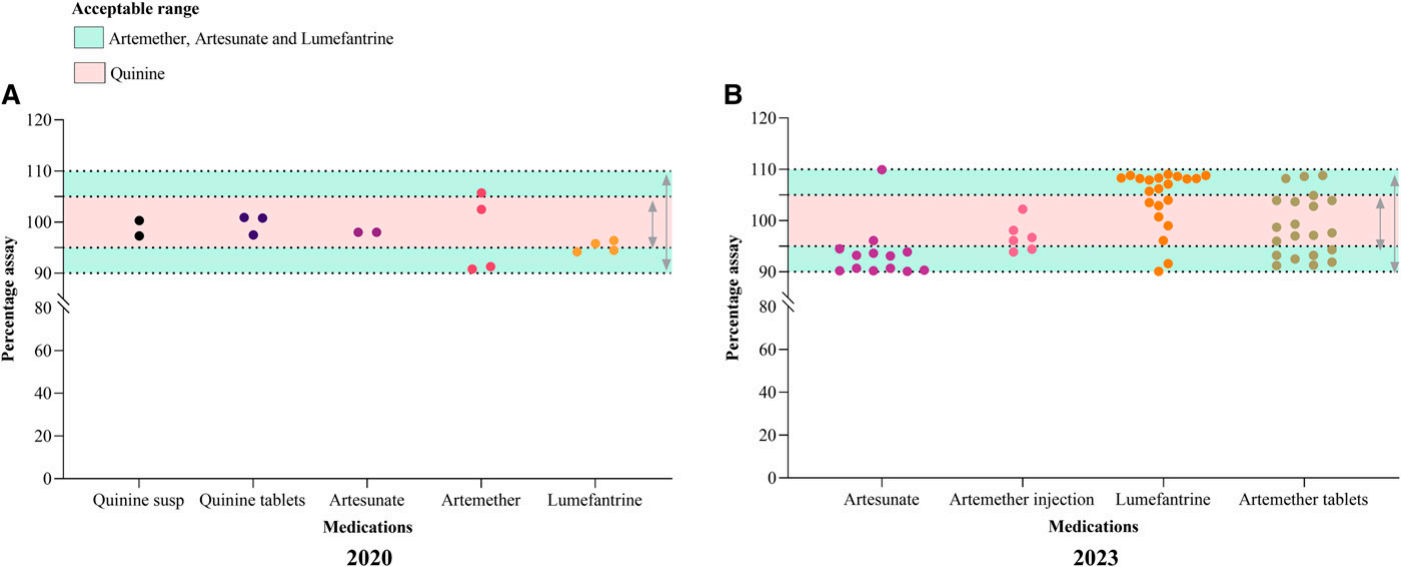
Amount of active pharmaceutical ingredient, expressed as % of the labeled amount for antimalarial medicines for (**A**) samples collected in 2020 and (**B**) samples collected in 2023 (*N* = 51). susp = suspension.

The assay results for the antimalarial medicines tested are shown in Figure 3. The amount of active pharmaceutical ingredient as a percentage of the labeled amount for all samples complied with the pharmacopeial requirements of 90–110% for artesunate, artemether, and lumefantrine and 95–105% for quinine (Figure 3).

## DISCUSSION

This study investigated the quality of antimalarial medicines present in the Tanzanian market, using the PMS approach. Ensuring the quality of antimalarial medication is paramount for safeguarding public health, given the substantial malaria burden prevalent in a significant portion of the country.^2^ These overarching findings suggest that antimalarial medicines in the Tanzanian market generally meet acceptable quality standards regarding physical and chemical characteristics per pharmacopeial monograph. However, a concerning aspect emerged, as approximately half of the samples collected from distribution outlets deviated from the TMDA labeling guidelines regarding product information.^23^

This study revealed that only approximately one-tenth of all collected antimalarial medicines were domestically manufactured, indicating a low manufacturing capacity and high dependency on imported medicines, primarily from India. This aligns with previous data highlighting the low market share of domestic industries in Tanzania.^24^ Overreliance on imported medicines threatens public health, particularly in emergencies such as the COVID-19 pandemic, when disruptions in the supply chain led to reduced accessibility of essential medications.^25^ There is a pressing need to attract investment in domestic manufacturing, particularly for medicines targeting poverty-related diseases.

The geographic distribution of the samples in this study indicated that more samples were collected from the Tanga and Dar es Salaam regions. In contrast, the lowest proportions were collected from the Kilimanjaro, Dodoma, and coastal regions. The number of samples reflects the availability of medicines at the time of collection, possibly influenced by the prevalence of malaria in the respective regions. Interestingly, fewer samples were collected in the Mtwara and Kagera regions, where malaria prevalence was greater than in Tanga and Dar es Salaam.^2^ The presence of ports of entry at Tanga and Dar es Salaam may explain this. In addition, this may be attributed to sample collection in peripheral areas, where the supply of medicines usually remains low. Among the collected samples, ALu was more prevalent than the other antimalarial drugs, likely because of its status as the recommended first-line treatment of malaria.^26^

This surveillance revealed that approximately half of the collected samples failed to meet the labeling requirements in the TMDA guidelines.^23^ Compared with 2020, 2023 had fewer deviations, indicating an improvement. Nevertheless, the findings suggest a persistent trend in noncompliance over the years, as observed in a previous study,^19^^,^^21^ despite the regulatory directives provided to market authorization holders (MAHs). Specific discrepancies were observed mainly on package leaflets, where necessary information was missing. The risks associated with improperly labeled medicines include medication errors and poor adherence, with serious consequences for patients.^27^ In the artwork, the products found in the market had some deviations including color, font size, and the addition or omission of some information compared with approved labeling orientations. The inconsistency in the artwork could reduce confidence and trust in the quality of the drugs by the community. Some of the products in the market had a labeled shelf life beyond the approved one in the registered product, implying that the community may unknowingly be consuming expired medicines. Similar findings were reported in a study done in Nigeria.^28^ The present study suggests potential negligence by MAHs regarding labeling or the challenges faced in complying with the labeling requirements of various regulatory authorities for the same products. Considering the crucial role of product information in prescribing, dispensing, safe use, and storing of medicines, our results suggest an urgent need for regulatory directives.

Remarkably, all the samples collected conformed to the screening and confirmatory test requirements. The findings differ from those of previous studies, which reported poor-quality antimalarial drugs in Tanzania.^16^^–^^21^ This indicates a notable improvement in the quality of antimalarial medicines circulating in the Tanzanian market over years of implementing the PMS program. Stringent procurement conditions may contribute to this improvement, including sourcing from prequalified WHO suppliers applicable to antimalarial medicines procured by government agencies. However, it is noteworthy that government-procured medicines constituted a minority of the sample in this study and were distributed only to public institutions. The conformity of all samples, irrespective of their sources, underscores the robust enforcement of rules and regulations governing the quality of medicines in the country. This enforcement positively impacted manufacturers’ adherence to good manufacturing practices, compliance with regulatory requirements by MAHs, and adherence to regulatory requirements, including storage conditions, by medicine distribution outlets. These results contradict the findings of previous studies from Malawi,^13^ Nigeria,^25^^,^^29^ and the Democratic Republic of Congo,^15^ which reported poor-quality antimalarial medicines circulating in the markets.

The presence of antimalarial medicines of acceptable quality in the country strengthens efforts for malaria control including prevention campaigns, treatment programs, and drug distribution. Antimalarial drugs with acceptable quality are essential for reducing disease transmission, preventing drug resistance, and achieving SDG number 3.3, which aims to eliminate malaria epidemics by 2030.^3^

Tanzania has recently confirmed partial artemisinin resistance in the northwest region of Kagera, and efforts to contain the spread are ongoing.^30^ Poor quality of antimalarial drugs is one of the potential drivers of drug resistance. The absence of poor-quality antimalarial drugs suggests that this factor may not be a potential driver of resistance development in Tanzania. On the contrary, recent reports indicate that the quality of 18.9% of ALu in Uganda,^31^ where artemisinin resistance is also prevalent, is substandard.^32^ The findings of this study are crucial to inform continuing efforts aimed at mitigating artemisinin and partner drug resistance in Tanzania and beyond. These findings can motivate regulatory decision-making and policy formulation, guiding efforts to strengthen drug regulation, enforcement, and surveillance systems, leading to improved oversight and ultimately safeguarding of public health through promoting access to high-quality medicines.

Our findings also provide critical reassurance to healthcare providers, regulatory agencies, and the public regarding the safety and efficacy of these life-saving medicines. This promotes trust in the healthcare system and fosters confidence in the treatment of malaria, ultimately leading to improved patient outcomes and reduced morbidity and mortality.

As a limitation of this study, confirmatory analysis for SP samples was not done because there were no samples eligible for the tests. This may have limited the generalization of our results, considering that poor-quality SP antimalarial medicines were prevalent in previous studies.^16^^–^^20^ However, it is worth noting that SP is currently not the treatment policy for malaria and it is restricted only to intermittent preventive treatment in pregnancy with limited availability in the market. The strengths of this study include the involvement of samples from many regions (15 regions) and sampling of the entire supply chain throughout the year, which indicates that the findings reflect real-world situations.

## CONCLUSION

The findings of this study indicate that the antimalarial medicines circulating in the mainland Tanzanian market meet quality standards. A persistent trend toward deviation from PIR requirements warrants an urgent need for regulatory directives. Continuous monitoring of antimalarial medicine quality is advocated.

## Supplemental Materials

10.4269/ajtmh.24-0145 Supplemental Materials
