# Supplementary material for: A Comprehensive Assessment of Quality of Antimalarial Medicines in Mainland Tanzania: Insights from Five Years of Postmarket Surveillance
Source: Am J Trop Med Hyg. 2024 Oct 1;111(6):1215–22. doi: 10.4269/ajtmh.24-0145 (PMC11619501; doi:10.4269/ajtmh.24-0145)
Supplement: Supplemental Materials [file tpmd240145.SD1.pdf]

Supplementary Table 1: Number of samples collected from each region

	Samples collected from each Region (n)															
**Type of medicine**	**TANGA**	**KAGERA**	**MWANZA**	**IRINGA**	**ARUSHA**	**KILIMANJARO**	**MARA**	**KATAVI**	**MBEYA**	**NJOMBE**	**MTWARA**	**DODOMA**	**PWANI**	**MOROGORO**	**DSM**	**Total**
Artemether 20 mg + Lumefantrine (tablets)	22	20	24	26	19	27	27	26	22	18	16	12	8	10	41	318
Artesunate 60/120 mg- (injection)	9	9	7	8	9	6	8	11	8	7	8	5	3	5	14	117
Artemether 80 mg (injection)	4	5	6	2	1	4	6	6	4	1	0	0	0	0	7	46
Qunine tablets	3	2	4	3	1	0	1	1	2	0	0	1	1	1	10	30
Qunine suspension	3	1	3	0	1	0	0	0	1	0	1	0	0	0	5	15
**Total**	**41**	**37**	**44**	**39**	**31**	**37**	**42**	**44**	**37**	**26**	**25**	**18**	**12**	**16**	**62**	**526**

Note: All the samples from the port of entry were collected in Dar es Salaam where the main port is located
